# Causes of dropout from health insurance program: An experience from Lumbini Province, Nepal

**DOI:** 10.1016/j.dialog.2023.100150

**Published:** 2023-08-02

**Authors:** Devaraj Acharya, Krishna Bahadur Thapa, Bhagawoti Sharma, Mohan Singh Rana

**Affiliations:** a Bhairahawa Multiple Campus [TU], Siddharthanagar, Rupandehi, Lumbini Province, Nepal; b Sanothimi Campus [TU], Bhaktapur, Bagmati Province, Nepal; c Mahendra Multiple Campus [TU], Nepalgunj, Banke, Lumbini Province, Nepal; d Mahendra Multiple Campus [TU], Ghorahi, Dang, Lumbini Province, Nepal

**Keywords:** Defaulter, Experience, Health insurance, Non-renewal, Universal health coverage

## Abstract

The Health Insurance Program (HIP) in Nepal is experiencing low enrolment and high dropout rates, but the causes of these issues have remained unknown. This study aimed to explore the causes of dropouts of the HIP implemented by the Health Insurance Board, Nepal.

We employed an exploratory qualitative research design. We purposefully selected the informants for the data collection who had previously enrolled and currently not renewed their insurance scheme. We gathered qualitative information from 16 in-depth interviews, four key informant interviews, and four focus group discussion in Palpa and Bardia Districts of Lumbini Province, Nepal. The qualitative data were analyzed using thematic analysis.

We identified two major themes and nine drop-out-related sub-themes. These were: unnecessary health insurance; negligence to renew; unable to pay the contribution amount; poor cooperation between institutions as well as insurees and insurers; limited coverage and ceiling amount; rigid processes to receive health services; health professionals' behaviors; poor quality healthcare services; inadequate information. Dropout-related factors were associated with personal or individual factors and institutional or policy-related (process-related) factors. The major causes/reasons for dropout include lengthy procedures, poor quality and unsatisfactory services, a lack of knowledge on health insurance norms and procedures, and health professionals' behavior towards insurees during treatment.

Information, education, and communication programs related to health insurance are still necessary to make the insurees familiar with the insurance systems and its processes. These factors could be taken into account by policymakers while planning interventions to minimize the low enrollment and high dropout.

## 1 Introduction

Health insurance is regarded as a sustainable financial strategy for reforming the healthcare system [1]. The healthcare systems of many developing and developed nations include health insurance. The Constitution of Nepal (2015) stated that everyone has the right to free basic health services and that no one shall be denied access to emergency healthcare services [2]. Moreover, the Government of Nepal (GoN) has committed to ensuring that the Sustainable Development Goals (SDGs) and Universal Health Coverage (UHC) targets that are expected to be achieved by 2030 [3]. In order to provide access to healthcare as per the targets, WHO member nations made a commitment developing a sustainable health funding system in 2005 [4]. Additionally, the WHO predicts that the catastrophic expenses associated with accessing healthcare services cause poverty to rise by around two percent annually [5]. Funding the healthcare system is a global priority for all countries, especially emerging ones, due to these financially catastrophic issues [6]. In developing nations, the majority of healthcare expenses are paid out-of-pocket (OOP) [7]. By managing sustainable financing for universal health coverage (UHC), the WHO and other developmental organizations developed a solution and member states endorsed it. Through a sustainable finance framework, the UHC ensures that high-quality healthcare is provided without economic burden [8]. The sustainability of health insurance schemes depends on the availability, high quality, and responsiveness of healthcare services [1].

It was noted that participation in national health accounts by individuals must be required to maintain sustainable financial management. Health insurance may be able to improve an access to healthcare by reducing financial barriers, raising enrolled participation in decision-making, and offering contractual agreements based on quality standards [9]. It was further thought that effective implementation of health insurance programs (HIP) would enhance the provision of efficient, competent, fair, and financially comfortable healthcare to the people [10]. In light of these realities, the GoN introduced HIP in 2016 in the initial phase in the districts Kailali, Baglung, and Ilam. Almost all districts and local municipalities are now covered by the insurance program [11]. When drop-out/non-renewal rates are rising, enrollment and retention/renewed rates are exceeding expectations. It was further noted that dropout rates ranged from 38 to 67% in several districts [12].

As per the current provision, a five-member family needs to pay 3,500.00 Nepali Rupee (NRs.) for enrolment that covers up to NRs. 100,000.00 and each additional member should pay extra NRs. 700.00 that covers NRs. 20,000.00 extra up to the maximum ceiling of NRs. 200,000.00 [13]. According to Health Insurance Board, nearly one-third of the total enrolees did not renew their insurance scheme in the fiscal year 2021/22 [13]. It is interesting that individuals wanted to join the HIP [14], and the majority of households (88%) desired enrollment [15], if they can receive quality services, they want to spend nearly twice as much as their present contribution amount [16]. However, the question of why they chose not to enroll and why they did not renew their insurance remains unanswered. We were unable to find any research on how people view health insurance and the reasons they are dissatisfied or do not renew. After a thorough discussion with participants in Lumbini Province, Nepal, the study seeks to address the gap. The study aims to answer the question- how did people experience/perceive health insurance with reference to causes of dropout?

## 2 Methods

### 2.1 Research design

An exploratory qualitative research design was employed. The focus of the narrative inquiry was to explore the participant's experiences, feelings, and perceptions toward the program. The method of narrative inquiry was deemed appropriate for this study because it has the potential to capture the essence of participants' experiences by providing a thorough account of their perceptions, emotions, and attitudes toward the program [17]. Narratives are a powerful tool for understanding the human experience. Experiences shaped by the construction and reconstruction of personal stories. Narratives record the experiences [18]. People shape their experiences by stories [19]. It is particularly useful for exploring the lived experience of a condition. We believed that, in narrative inquiry, participants share their stories about their experiences with health insurance. These stories are then analyzed to identify themes and insights [20]. The analysis of these stories can help researchers to understand the different perspectives of people with health insurance, and to identify the hidden assumptions that people have about health insurance. The narratives of participants are also assessed from different perspectives [21]. This can be done for data triangulate to ensure the reliability and validity of information [22].

#### 2.1.1 Study site

In Lumbini Province, there are 12 districts, including six in the Terai and the same in the Hill regions. Two districts were purposively selected: one from the Terai and one from the Hill. The reason for selecting these districts was that they were the first to implement the health insurance program [23].

#### 2.1.2 Study participants and selection procedure

Altogether 16 participants were selected for in-depth interviews (IDI), which was more than the minimum required number for qualitative research [24] though it was determined by data saturation. In both the rural and urban municipalities, four participants were purposefully chosen for IDI from each district. As a result, eight participants were selected equally from the districts of Palpa and Bardia. Similarly, two focus groups from Palpa and equally from the Bardia district were formed as part of four focus group discussions (FGD). The characteristics or eligibility of FGD participants were as same as IDI participants. Enrollment assistants (EA) were chosen for the key informant interviews (KII) because they are more responsible for making enrollment and are more likely to hear complaints from insured people. A total of four KIIs, two from each district, were conducted for data triangulation to increase the validity and reliability of the data [25].

In order to get relevant information from various areas and issues with different residential and environmental conditions, participants were selected from different fields. The participants were chosen based on different factors such as gender, caste, occupation, area of residence, and level of wealth, and they were anonymized to ensure their privacy and confidentiality.

#### 2.1.3 Data collection tools

Guidelines for IDI, FGD, and KII were employed to collect data. In order to validate these guidelines, a mock session was held in Palpa and Bardia Districts from October 24 to 29, 2021. The samples used for the mock session were not used for the final data collection. With all the investigators, the study tools were finalized after a comprehensive discussion with the research experts.

#### 2.1.4 Data collection procedure

After completing all required administrative processes, the research teams were sent out to gather data in their respective districts. Written consent was taken prior to the interview or discussion. Altogether, 25 participants were selected for the four FGDs, while the IDI and KII were conducted individually at convenient places for them. Researchers deployed in Palpa and Bardia, were fluent in local (Magar and Tharu) languages. Interviews and discussions were audio-recorded upon their permission to record. Data collection was placed in Bardia from December 2 to 29, 2021, and in Palpa from January sixth to February 17, 2022. Measures of quality assurance were followed during the data collection such as transcription of notes were made right after completing the interviews and discussions [[26], [27], [28], [29]].

#### 2.1.5 Data analysis

The primary sources for the data analysis were the IDI, FGD, and KII all were recorded and transcribed. The participants' actual language was used to translate the raw data into text. The transcription was thereafter carefully examined. The purpose of the study served as a guide for the analysis. The study team applied the basics of categorizing, coding, and displaying qualitative data [29]. The transcript was frequently read over before codes were created. Two researchers were involved in data coding. The code was given a name in the initial stage using the participants' original words. The original text then concentrated on the participants' perceptions and lived experiences [30].

Following the creation of the code, related codes were combined into sub-themes and themes by carefully studying the narratives. These topics were combined using "catchy verbatim," and the theme or verbatim was then translated from the Tharu, Nepali and Magar languages into English with regard to the objective. A thorough examination of the transcripts, codes, and the themes were followed by the creation of the thematic analysis [31]. The results were contrasted with those of previous studies. A comprehensive discussion was followed by a summary of the study's findings [32].

During analysis and discussion, we applied the following codes to the verbatim [See Table 1]:

**Table 1:** Code used for participants for their verbatim.

Particular	Attributes	Code used	Remarks
District	Palpa Bardia	P B	
Interview	Key informant interview In-depth interview	KII IDI	
Discussion	Focus group discussion	FGD	
Participants age	Age in years	–	Completed age
Gender	Female Male	F M	

Note: B-KII-40-M refers to the informants of Bardia District involved as a Key Informant Interview age of 40 years and male.

#### 2.1.6 Data quality and assessment

The study team received training on the concerns with the quality of qualitative research. To assure the data quality in qualitative data, we considered quality issues as applicable and possible before and during the data collection [33,34].

### 2.2 Ethical/safety issues

Throughout the research process, the ethical fundamental norms of beneficence, justice, and respect for informants were respected and followed [35,36]. The guidelines for ethical issues [37] and national ethical procedures for health research [38] have been maintained and adopted while accomplishing the research. Moreover, Nepal Health Research Council reviewed and approved the research proposal [NHRC Ref # 1014, 27 Oct 2021; Registration # 582/2021 P].

## 3 Results

### 3.1 Background of the informants

Participants for IDIs FGDs, and KIIs were the three types of participants we interviewed and discussed. There were 16 IDI participants, four KII participants and 25 FGD participants within the four groups. Information was collected from 45 research informants. The mean age of the participants/informants was 42.8±11.5 years and the median, mode, and range were 44, 48, and 47 years respectively. Participants were from the age of 24 to 71 years. Almost one out of ten (11%) of the participants were illiterate. The majority (58.7%) of participants were females, the majority (64.4%) of them were from joint families, and their main occupation was agriculture (40%) followed by domestic work (20%), business and services equally (15.6%).

### 3.2 Causes of dropout

After carefully analyzing the data (transcript), we came up with the themes. The following causes/problems were identified as the main reasons for dropouts [See Table 2]:

**Table 2:** Themes, sub-themes and codes used for causes of dropout from health insurance program.

Themes	Sub-themes	Codes	Remark
Individual or personal related factors	Unnecessary	The feeling of healthiness, perceived of secured, feeling of no susceptibility to diseases, and investment in health insurance is considered as wastage	
	Negligence	Less priority towards insurance, negligence	
	Inability to pay	Unable to pay, flat system to pay, no provision of payment on the installment basis	
	Poor co-operation	Poor communication between enrollment assistant and insurees, poor interaction	These are common in both policy and personal related factors
	Conduct of health professionals	Ignorance by health workers, mis-behave towards the patients and insurees, not properly listening to patients	
	Low quality services	Unavailability of recommended drugs, long waiting at registration/diagnostics/consultation,	
	Insufficient information	Unaware of scheme, unfamiliar with referral process, limited knowledge of coverage and procedures	
Institutional or policy related factors	Poor co-operation	Poor interaction, lacks of institutional cooperation (service providers and regulating body)	
	Conduct of health professionals	The same patients are respected at the private clinics but ignored at the government hospitals, come tomorrow, ignorance, and poor attention towards patients	
	Low quality services	Unavailability of medicine, discrimination, long waiting times, rigidness, poor arrangement of siting waiting places,	
	Insufficient information	Unknown about health insurance and processes, limited information about health care providers, procedural gaps	
	Limited coverage	The limited ceiling of NRs. 100000, limited medicine available, and limited health facilities listed under the scheme.	
	Annoying process	Rigid referral process, time consuming in registration and other processes, multiple registrations (test/diagnostics, account, consultation)	

#### 3.2.1 Unnecessary

It is true that without any prior information, people get a disease or illness. Prioritizing the issues, one is currently dealing with is a common practice. An individual might not appreciate the benefits of healthcare until they get sick or injured. Participants indicated that after subscribing to the health insurance program, they never used the health services because they did not require medical treatment for any conditions. They decided not to renew their insurance, since they believed that the health insurance program was useless for young people. For those taking frequent medicine for a chronic condition, they believed it to be essential. They may believe they can spend the contribution amount, which must be paid to continue the program, in another way, such as buying household items for usage on a daily basis. Some of the participants stated that managing other things besides their health was their top priority and not their health.

**Table:** 

*Insurees used to remark, "We do not need medicine, so why do we enrol in health insurance?" They also say, "Individuals see themselves as healthy, therefore they think it is meaningless to pay contribution amount for health insurance".* -[P-KII-26-F]

#### 3.2.2 Negligence

People by nature tend to prioritize the things that are going on right now rather than the things that will happen in the future. Even if they might not require the services all year round, not all insured individuals may require medical treatment. Therefore, they forget to renew their plan. Participants stated that because they had not used the treatments covered by their health insurance plan, they had forgotten to renew them. One of the participants complained that neither had we phoned the EA to renew our plan nor did she call us. Furthermore, they claimed that the program belonged to them and that it was their duty to renew it, but owing to their negligence, they forgot to renew their insurance scheme.

**Table:** 

*I overlooked the renewal date. I didn't phone the enrollment assistant to renew the insurance plan, and neither did the enrollment assistant ask me to do so.* -[P-IDI-28-F]

#### 3.2.3 Inability to pay

Economic survey shows that more than 15 percent of Nepalese people are living below the absolutely poverty [39]. Therefore, people may not be able to pay the contribution amount required to renew the health insurance plan since they are still having trouble feeding themselves. One of the participants claimed that they could only manage their food on a daily basis if they found labor work. If not, they were even unable to manage their daily expenses. They might not be able to afford the renewal of the scheme in such a case. The participants also asked the responsible person if they may pay the contribution amount in monthly or trimonthly instalments, as that would be preferable. They said that paying in one lump sum each year was too expensive for them financially. An enrolment assistant expressed:

**Table:** 

*People are poor where I live. They were unable to contribute the required amount. Additionally, the majority of those with insurance complain about the hospital services and level of care. According to the benefits package, the hospital did not offer medicine. According to patents, hospitals used to exclusively offer "cheap medicine," not expensive medications.* -[B-KII-49-F]

#### 3.2.4 Poor cooperation

To make enrolling the household at the local level easier, EAs have been assigned at the local level. They only work on an incentive basis and are not paid on a monthly basis at the ward level. Therefore, EAs may have another employment for their livelihood and may be busy with their paid job; as a result, they may perceive the position of EA as a secondary job or as having lower priority. One of the research participants said they had not called the enrolling assistant to renew the plan and she had not either reminded them to do so. Similar situations occurred when enrolling helpers visited their homes while they were away. As a result, it was revealed that the EAs and the insurees failed to work together. Not only that, it was noticed that there was poor cooperation between institutions too.

**Table:** 

*I didn't have any money when the enrollment assistant arrived, and when she didn't come when I had money.* -[P-IDI-48-F]

#### 3.2.5 Limited coverage

A 5-member household is often required to pay Nepali Rupees (NRs.) 3500 (US $27) for enrollment, which covers up to NRs. 100000 (US $780) with the maximum ceiling of NRs. 200000. Additionally, there are various rules and guidelines for the services, insurance, and payment process. We discovered that the majority of participants saw registering for health insurance as the best, most ideal and long-lasting solution to any health issues, including treatment and medical services. However, the truth, is far different. One of the participants said that the health insurance coverage is quite limited and only applies to a few services, and that the coverage items and ceiling amount need to be re-evaluated. The terms and conditions of the health insurance program state that insufficient coverage of up to only NRs 100,000.00 was noticeable while receiving healthcare and medical services. The major deterrent for not renewing was the ceiling amount is just NRs. 100,000.00. Participants further stated that because they were unable to receive the treatments they desired and had to pay for their medications, they believed that it was worthless to continue the program because it provides inadequate coverage of the costs as well as healthcare services.

**Table:** 

*We can afford up to NRs. 100,000.00 but not more than that. We had to purchase expensive medicines from a private pharmacy because only "limited and basic medicines" were provided under the insurance scheme, but they were not costly. I paid the contribution/premium amount for free medicine and services. All services should be free of cost and should not limit it.* -[B-FGD-41-M]

#### 3.2.6 Annoying process

There are some requirements for receiving healthcare services after enrollment that call for specified procedures that insured individuals should adhere to. For example, the primary (first) service point should be a government health facility, and if referral services are required, the insured person should obtain a referral slip from the first service point. Participants complained that the provision was unsuitable, inappropriate, impractical, time-consuming, and complicated to receive the services.

**Table:** 

*We are required to pay 600 Nepali Rupees in travel expenses for just a 60-rupee prescription. Since receiving services was so difficult, I made the decision to stop.* -[P-FGD-44-M] *The referral process is the one that patients find the most annoying. The majority of individuals under the scheme did not receive any medicines. They have a lengthy wait. They finally decide to quit the insurance scheme.* -[P-KII-24-F] *We must allocate one day to the referral process, one day to the check-up, and one more day to the clinical diagnosis. As a result, the procedure takes a long time. All the treatments can be finished in the private clinic in just one day. I gave up since I didn't like it.*-[B-IDI-48-F]

#### 3.2.7 Conduct of health professionals

Insured people do not pay cash to have an insurance card, hospitals used to submit claims to the health insurance board, and healthcare professionals do not give priority to the insurance cardholders. Health professionals do not prioritize insured people because they must ask the health insurance board to obtain prompt payment from the insured instead of paying the insured.

**Table:** 

*Even for broad counsel, doctors used to say, "come tomorrow." In addition, they send us "here and there." It is quite annoying. They do not thoroughly observe our concerns. However, the same doctor used to respect us in their private clinic.* -[B-IDI-52-M] *Doctors do not care about us seriously. I lost my husband due to the negligence of the doctor. I wanted to terminate the scheme.* -[P-IDI-65-F] *Doctors used to treat uninsured patients first and insured patients later. During treatment, doctors insulted us while receiving services under the insurance program and I felt discouraged. I therefore made the decision to reject the scheme.* -[P-IDI-60-M] *Under the insurance plan, we were unable to receive any services. Health professionals acted in a doglike manner. They used to treat us harshly. Doctors would advise, "come the day after tomorrow".* -[B-IDI-58-M]

#### 3.2.8 Low quality services

Despite being insured, insurees complained that they did not receive high-quality service. Participants stated: ‘you cannot get the service, you need even after waiting in line for a very long time, renewing your insurance is not considered necessary’. Participants also reported that numerous individuals had to undergo health examinations immediately, and it was observed that the patients received substandard medical care. Due to a shortage of high-quality healthcare, health insurees were found to be unwilling to renew their insurance scheme. Participants stated that only a few fortunate insured individuals could obtain medications as per a physician's prescription.

**Table:** 

*Most patients have to purchase medicines from private pharmacies, which are more expensive than expected. They finally decide to give up the plan.* -[P-KII-24-F] *Even paracetamol was not available to us under the program. Doctors used to diagnose patients based on their social background. You can obtain services if you have access or are in an elite class. I was unable to obtain the medicine that the doctor had prescribed, but another patient who had access to it (perhaps due to politics) was able to receive medicine at the same time.* -[B-IDI-64-M]

#### 3.2.9 Insufficient information

Although health insurance has not been introduced in Nepal for a long time, due to a lack of awareness about insurance, both those who have and have not been insured have many complaints about the health insurance program. Various public objections about the referral system health insurance program have been caused, in part, by a lack of awareness about the healthcare program, the cost of services and medications under it, as well as other procedural information. Each stage and process in a health insurance scheme is distinct. Because people are unaware of the procedures, insurees frequently faced problems that affect the success of the overall health insurance system. Additionally, it is obvious that insurees misunderstood the health insurance program because of their lack of prior knowledge of health insurance.

**Table:** 

*The majority of insurees are not aware of how to best utilize the scheme's services. They must complete a complex referral process, which causes hesitation in them, and they ultimately choose to exit the program.* -[P-IDI-47-F] *The public do not have adequate understanding and information on health insurance and its process. Due to the lack of drugs covered by the health insurance plan, the majority of the insurees did not renew their plan. The hospital offered "cheap medicine," but not "expensive medicine," which had to be purchased outside. The medical retailer would fear losing their jobs if the program is successful, thus they used to persuade people that "the health insurance scheme is worthless".* -[B-KII-34-M]

## 4 Discussion

Major reasons for not renewing the health insurance program have been identified based on the findings. First, the factors that are related to individuals include carelessness, their economic ability, a lack of cooperation on the part of insured individuals and EAs, and the conduct of healthcare professionals toward patients and insured individuals. Second, the institutional or policy related factors primarily connected to health facilities or policy are inadequate drug coverage, inadequate coverage and selective services/facilities, a time-consuming or drawn-out process during service utilization, and a lack of adequate information regarding the process and standards of health insurance. Some policy or process-related factors based on health insurance implementation guidelines include low cooperation between the Health Insurance Board and service providers (i.e., government hospitals, community hospitals, charity-based hospitals, and private hospitals), low or poor quality of health services, rigorous process, lengthy waiting times for check-ups and diagnostic tests, and health workers' behavior are identified as dissatisfaction issues contributing to high dropouts. These process-related issues are also thought of as system-related factors that the formal legal system or policy should address.

People cannot be perfectly satisfied, yet there can be levels of satisfaction and dissatisfaction. The majority of studies have found quality health care as a strong predictor of the continuation of a health insurance program. High dropout rates and unhappiness with the health insurance program are caused by poor service [40]. From a policy perspective, it was proposed that it is crucial to carefully assess the fair outcomes of community-based health insurance programs before deploying them to further the mission of universal health coverage [41]. Strict regulations governing insurance programs, an ineffective legal and legislative framework, and limited benefit packages all contribute to high dropout rates [7] which are almost similar to the findings of the study.

As our findings show, people tend to discontinue participating in community-based or social health insurance, however some do so voluntarily and others are forced to. Only some insured 'lucky/privileged' members can get healthcare services, which causes the insured members dissatisfied with the health insurance program [40]. All three factors: individual, governmental, and procedural factors are accountable for unhappiness and dropout. It has been determined that the availability of drugs, insured members' good health, healthcare providers' behavior toward them, and rented accommodation have a negative impact on whether a health insurance program will be renewed [42,43]. However, experiences and perceptions of poor quality healthcare services are identified as the most influencing factor for dropout [40,[44], [45], [46]]. On the other hand, it has been proven that coverage limits are the factors in India's low retention rate for health insurance [47] which can be categorized as institutional or policy-related issues, which is almost similar to our findings.

In general, it is impossible to satisfy every insured person. However, it should be remembered that the insurance program's sustainability would be uncertain without the satisfaction of the insured. According to a study from Bangladesh, one-third of the members were very satisfied with the comprehensive healthcare services offered under the insurance program [1]. Another systematic review came to the conclusion that system, community, interpersonal, and individual factors all contribute to the purchase and renewal of health insurance [8]. Similarly, people receiving health care services through their insurance policy were more willing to renew the policy than those who did not receive such services [48]. Another study revealed a favorable relationship between the renewal of the insurance program and the use of healthcare services covered by the plan. By managing the pre-pay system or financial protection that may be influenced by dropout, access to healthcare for all is accepted worldwide [49]. The insured members' opinions on Ghana's insurance program and the standard of medical care were determined to be unfavorable [50]. The level of quality service has been conclusively identified as the most important aspect in determining whether a health insurance program will be renewed [40].

The length of registration, appointments, access to medical facilities, insurance knowledge, and officials' decision-making were all directly associated with the decision regarding the continuation of insurance programs [6]. More than one-fifth of the overall study participants dropped out of the health insurance program, according to a study from Vietnam. Younger age, lower education, being small traders, jobs in the informal market, and being not poor were associated with dropout [51]. The continuation of the insurance program may be influenced by a few other factors connected to health service subsidies. According to regulations and acts, basic medical services, critical illnesses like cancer, kidney disease, Parkinson's disease, and sickle-cell anaemia, patients with a red card, and people over the age over of 70 years are all provided with healthcare services without any cost in some contexts in Nepal. These provisions can cause individuals to drop out of the health insurance program. Similar findings have been reported, with social support cards noted as the prime reason for withdrawals in Sudan [52].

Different countries and circumstances may have different dropout factors. An experience from Ghana demonstrated that between the years 2008 and 2012, dropout rates ranged from 7 to 35 percent. In addition, the incidence of illnesses, a minimal benefits package, and substandard services were the primary causes of dropout [46]. High dropout rates were mostly caused by female family heads, rare sicknesses in the family, lower educational status, and poor quality health care [45] which are almost comparable to the study. Most of the studies showed the perception of poor quality healthcare services is one of the main contributing factors to the non-renewal of health insurance [40]. Our findings echo those from Ghana where the dropout rate ranged from 41 to 53 percent between the years 2014 and 2016, and males and indigenous (poor) people were the major contributors [53].

In Ethiopia, a significant result that was comparable to our study was observed. Significant predictors of dropout included old age, education level, family size, perceived low-quality services, lack of trust in services and healthcare facilities, behavior of healthcare professionals, and coverage package included in the insurance scheme [54]. Another study from China explored that those who perceived themselves as healthy and those who used healthcare services unsatisfactorily were more likely to discontinue the insurance program [43]. However, such perception is not directly linked to significant dropout rates. Instead, being unable to pay the premium amount has been linked to dropout in several low- and middle-income settings [55]. Similarly, non-renewal rates in Uganda are closely related to the status of household wealth [56] and one of the causes of dropout is also unable to pay the contribution amount [57,58].

### 4.1 Strengths and limitations of the study

The research was conducted in both mountain and terai covering both rural and urban areas therefore it is expected that it covered a wide range of information from geographic and cultural diversity. Both districts had comparatively longer experience in health insurance implementation. Based on that, it is expected that the information we collected were more reliable as well as authentic. Moreover, we collected data from different participants from different sources such as in-depth interviews, focus group discussion, and key informant interviews. We presented the themes were triangulated from different sources such as IDI, FGD, and KII. Therefore, it can be assumed that all these themes/sub-themes are the most powerful evidences for policy making too.

Despite all the strengths, there are some limitations too. Findings from the study may not apply to other contexts or districts because it used non-probability sampling procedures in the districts of Palpa and Bardia. Therefore, the findings may or may not represent other districts and/or the whole country. The selection bias, knowledge bias, and even recall bias may persist with regard to participants, the research team, and even the study process itself. Since data analysis was carried out based on the data gathered from the participants, data credibility is subject to the information and responses provided by them. Despite the fact that information was gathered from 45 individuals in various settings in accordance with the data saturation principle, some information might have been lost. The study team's experience may have had an impact on the data analysis, which could have affected the presentation.

## 5 Conclusions

In the general term, the standards of quality are satisfaction and happiness. Some factors such as mutual trust, cooperation, and accountability may help people feel more optimistic about the health insurance scheme. These elements ultimately encourage participation in and renewal of the insurance program. This study identified some variables that are responsible for dropout and other studies have supported our findings. The main causes that have been found to predispose are frustration ultimately causing them to drop out.

### 5.1 Individual or personal related factors

Following a thorough analysis of the data provided by participants and findings from earlier studies, we identified a few demand-side or personally relevant elements and difficulties. Some of them include people' perception of themselves as being healthy and not requiring need insurance, inadequate information, and ignorance about renewal dates, economic hardship, and poor collaboration with the EA. Therefore, there may be lower chances of providing quality healthcare to patients and clients. In the same way, individual differences also affect the behavior how they perceived and presented. These factors also affect the satisfaction or dissatisfaction as well as retention or dropout. The personal related issues come under this category.

### 5.2 Institutional or policy related factors

Some problems have to do with service providers that can be considered as policy-related issues. We noticed some problems with the supply side. Selective and limited coverage, drawn-out and complex processes, absence of efficient management (irresponsibility/unaccountability), poor communication situations (institutions to institutions), poor service quality, the infrastructure of health facilities, and service providers' behavior are some examples of supply-side or institutions-related factors. These factors also include the infrastructure of the health facilities such as if there are sufficient space to stay and comfortable seat to sit, patients may not complain about time. In the same way, sufficient human resources for health (HRH) also affects the quality of health services. In the context of Nepal, there may not be sufficient HRH which ultimately makes the clients unhappy and dissatisfied. Institution related issues come under this category.

Some factors are identified to be related to process and system or policy. They include lack of proper information, distance to the medical institutions, lack of collaboration between the implementing and regulatory authority and service providers, rigid referral procedure, limited and unfair coverage services, and uniform policy and terms and conditions. Some problems are mostly connected to the government's and insurance program's policies such as the absence of an integrated policy. Policy, act, regulations and process related issues are considered under this category.

In order to reduce the non-renewal rate by raising awareness, regulating/implementing agencies and service providers should communicate sufficient information to people. Moreover, high-quality services are required for the sustainability of the program. Policy makers could consider these findings while making intervention plans to overcome the high dropout rate.

## Funding

NHRC [Provincial Health Research Grant, Ref. 778-2078/79].

## Ethics approval statement

NHRC reviewed and approved the study proposal [NHRC Ref # 1014, 27 Oct 2021; Registration # 582/2021 P].

## Declaration of Competing Interest

The authors declare that they have no competing interests.
